# Integrating diabetes, hypertension and HIV care in sub-Saharan Africa: a Delphi consensus study on international best practice

**DOI:** 10.1186/s12913-021-07073-0

**Published:** 2021-11-15

**Authors:** Geoff McCombe, Sara Murtagh, Jeffrey V. Lazarus, Marie Claire Van Hout, Max Bachmann, Shabbar Jaffar, Anupam Garrib, Kaushik Ramaiya, Nelson K. Sewankambo, Sayoki Mfinanga, Walter Cullen

**Affiliations:** 1 University College Dublin, Dublin, Ireland; 2 Barcelona Institute for Global Health (ISGlobal), Hospital Clínic, University of Barcelona, Barcelona, Spain; 3 Liverpool John Moores University, Liverpool, UK; 4 University of East Anglia, Norwich, UK; 5 Liverpool School of Tropical Medicine, Liverpool, UK; 6 Shree Hindu Mandal Hospital, Dar es Salaam, Tanzania; 7 Makerere University, Kampala, Uganda; 8 National Institute for Medical Research, Dar es Salaam, Tanzania

**Keywords:** HIV, Healthcare utilization, Noncommunicable diseases, Integration, Service delivery, Sub-Saharan Africa

## Abstract

**Background:**

Although HIV continues to have a high prevalence among adults in sub-Saharan Africa (SSA), the burden of noncommunicable diseases (NCD) such as diabetes and hypertension is increasing rapidly. There is an urgent need to expand the capacity of healthcare systems in SSA to provide NCD services and scale up existing chronic care management pathways. The aim of this study was to identify key components, outcomes, and best practice in integrated service provision for the prevention, identification and treatment of HIV, hypertension and diabetes.

**Methods:**

An international, multi stakeholder e-Delphi consensus study was conducted over two successive rounds. In Round 1, 24 participants were asked to score 27 statements, under the headings ‘Service Provision’ and ‘Benefits of Integration’, by importance. In Round 2, the 16 participants who completed Round 1 were shown the distribution of scores from other participants along with the score that they attributed to an outcome and were asked to reflect on the score they gave, based on the scores of the other participants and then to rescore if they wished to. Nine participants completed Round 2.

**Results:**

Based on the Round 1 ranking, 19 of the 27 outcomes met the 70% threshold for consensus. Four additional outcomes suggested by participants in Round 1 were added to Round 2, and upon review by participants, 22 of the 31 outcomes met the consensus threshold. The five items participants scored from 7 to 9 in both rounds as essential for effective integrated healthcare delivery of health services for chronic conditions were improved data collection and surveillance of NCDs among people living with HIV to inform integrated NCD/HIV programme management, strengthened drug procurement systems, availability of equipment and access to relevant blood tests, health education for all chronic conditions, and enhanced continuity of care for patients with multimorbidity.

**Conclusions:**

This study highlights the outcomes which may form key components of future complex interventions to define a model of integrated healthcare delivery for diabetes, hypertension and HIV in sub-Saharan Africa.

## Introduction

While HIV continues to have a high prevalence in sub-Saharan Africa (SSA) among adults, the burden of noncommunicable diseases (NCD) is increasing rapidly, in particular diabetes and hypertension [1]. Each year over three-quarters (28 million) of global NCD deaths and most premature deaths from NCDs (82%) occur in low-to-middle-income-countries (LMICs). Cardiovascular diseases account for most NCD deaths, 17.9 million people annually, followed by cancers (9.3 million), respiratory diseases (4.1 million), and diabetes (1.5 million) [2]. The International Diabetes Federation reported that the prevalence of diabetes in SSA is anticipated to double between 2010 and 2030 [3]. It is estimated that of approximately 650 million people in SSA, 10–20 million may have hypertension [4]. However, these estimations are based on scarce heterogenous studies and many countries in SSA still lack detailed up-to-date basic data on the prevalence of hypertension [5]. NCDs are important contributors to the burden of disease in countries at all stages of economic development. However, the Global Status Report on NCDs emphasizes that the negative impacts of NCDs are particularly detrimental to populations with high poverty such as SSA [6], as poverty exacerbates many health conditions [7].

In recent years, there have been rapid improvements in HIV care programmes in SSA. Substantial global investment in health services has strengthened physical infrastructure, laboratory capacity, health information systems, healthcare worker capacity development and promoting delivery of antenatal care, family planning and sexually transmitted infection (STI) management [8]. This has led to the expansion of and improvements in life-saving antiretroviral therapy (ART) which has greatly decreased HIV related morbidity and mortality [9]. Currently, about 65% of all people living with HIV (PLHIV) in Africa regularly access care with antiretroviral therapy [10]. However, this has resulted in an ageing population of PLHIV, with the population becoming more susceptible to NCDs, such as diabetes and hypertension [11]. In contrast, health service provision for NCDs in SSA remains poor and evidence on adherence to treatment and retention in care is limited with only 5–20% of people with diabetes or hypertension thought to be in regular care [12]. There is an urgent need to expand the capacity of healthcare systems in SSA to provide services for managing HIV and one or more NCDs concurrently.

Within healthcare systems in SSA, as concern about the management of NCDs among PLHIV grows, the infrastructure and lessons learnt from the HIV chronic disease management model are important resources for those hoping to expand NCD prevention, care, and treatment [13]. These include health services which are stand-alone and vertically delivered and have been combined with decentralisation and task shifting, allowing primary health centres to treat large numbers of patients with almost 70% of people living with HIV-infection in regular care [14]. Given the similarities between different chronic diseases (their effects on health and individual functioning share common pathways and outcomes), the healthcare systems, assessment tools to diagnose and manage patients, health professional capacity and implementation strategies developed to provide continuity of care for HIV in SSA means they can potentially be rapidly, efficiently, and effectively utilized to support services for NCDs, particularly hypertension and diabetes [13].

Potential benefits of HIV-NCD integration for the health system and patients include a reduction in duplication and fragmentation of services, which would increase efficiency of resource use and help patients remain in care by reducing costs and inconvenience for patients with multiple morbidities [15]. Furthermore, screening for NCDs within HIV care programmes can improve the identification of undiagnosed NCDs among patients living with HIV and also contribute to improved health outcomes [16]. Leveraging and adapting the existing HIV model to integrate with newly developing NCD services is key to achieving integrated care systems that are more convenient for patients. However, although a number of models of integrated HIV/NCD care in SSA have been established in recent years, the lack of evidence-based care models for scaling up integrated care makes it difficult for countries to develop effective and contextually appropriate policy and practice based strategies [17].

Given the importance of integrated care models to address the issues outlined above, the aim of this study was to determine consensus among experts on key components/outcomes and best practices in integrated service provision for the prevention, identification and treatment of HIV, hypertension and diabetes.

## Method

The study design utilised an international, multi-stakeholder e-Delphi consensus study over two successive rounds. A web-based system designed to facilitate the building and management of Delphi surveys (DelphiManager, http://www.comet-initiative.org/delphimanager/), was used for data collection. The Delphi consensus technique is a survey method designed to obtain the opinions of a group of experts on a topic, with each round providing input to the next [18]. This technique differs from other group decision making processes in four ways: utilising anonymity, iteration and controlled feedback, statistical group response and expert input [19]. The Delphi approach is an iterative consensus technique that presents a series of sequential questionnaires asking individuals to rank outcomes in terms of priority for inclusion in a key components/outcome set that should be used in interventions to optimise the prevention, identification and treatment of HIV, diabetes and hypertension in integrated services in the SSA context.

This Delphi consensus study used two rounds with participants being informed of the results of the prior round and allowed to revise their opinion based on those results. The goal was to achieve a pre-defined threshold of consensus. Key to the Delphi approach is the anonymity of participants. By ensuring that participants remain anonymous throughout the process, they are free to revise their opinion without fear of reputational harm or to refuse to revise their opinion without pressure from the group to do so [20].

Data were collected from the selected group of global expertson integrating diabetes, hypertension and HIV Care in SSA by using formal consensus methods, defined as “group facilitation techniques designed to explore the level of consensus among a group of experts by synthesising and clarifying expert opinions.” [21].

### Selection of experts

Consistent with the purposive sampling approach used by many Delphi studies [20], our sampling strategy focused on identifying potential experts on the topic of interest and inviting them to participate in the study. We identified potential experts through the ‘INTE-AFRICA’ consortium (*n* = 39) [22] and by contacting the corresponding authors of manuscripts from a scoping review we conducted on integrating care for diabetes, hypertension and HIV in SSA (*n* = 38). Expressions of interest to participate in the study were sought from the 77 identified experts.

Twenty-four participants returned an expression of interest form and were sent an email invitation that included information about the purpose and process of the study and a link to the online version of the questionnaire in DelphiManager. The size of Delphi panels can range widely and the 24 participants that agreed to participate in this study is within the 10–50 typically recommended [23]. We asked the experts to commit their participation for two planned Delphi rounds and informed consent was obtained from all participants. Participants were recruited between December 2019 and January 2020. Consistent with the COMET methodology, we included researchers, policymakers, and academics in our sample of experts to ensure a broad representation of opinions (Table 1). The establishment of this panel was overseen by the INTE-AFRICA Work Package 3 steering group which was responsible for providing advice, ensuring delivery of Work Package 3 project outputs. The Delphi panel participants received no financial incentive to participate in the study.

**Table 1:** Demographic characteristics of invited participants who expressed an interest in participating

Participant (***n***=24)	Location	Category	Completed Round 1 (***n***=16)	Completed Round 2 (***n***=9)
1	UK	Academic / researcher	Yes	No
2	UK	Academic / researcher	Yes	No
3	Malawi	Academic / researcher	Yes	Yes
4	Uganda	Academic / researcher	Yes	No
5	Tanzania	Policy maker	Yes	Yes
6	USA	Academic / researcher	Yes	No
7	Tanzania	Academic / researcher	Yes	No
8	UK	Academic / researcher	Yes	Yes
9	Tanzania	Academic / Physician	Yes	Yes
10	UK	Academic / Physician / Policy maker	Yes	No
11	Tanzania	Academic / Physician	Yes	Yes
12	UK	Academic / researcher	Yes	Yes
13	Uganda	Academic / researcher	Yes	Yes
14	Botswana	Policy maker	Yes	No
15	UK	Academic / Physician	Yes	Yes
16	Canada	Researcher	Yes	Yes
17	Uganda	Physician / Policy maker	No	No
18	UK	Academic / researcher	No	No
19	Uganda	Academic/ Physician	No	No
20	India	Academic / researcher	No	No
21	Nigeria	Academic / Physician	^a^No	No
22	UK	Researcher	No	No
23	Nigeria	Academic / Researcher	No	No
24	USA	Researcher	^a^No	No

^a^Participated in Round 1 but did not complete

### Round 1

Participants were asked to score the importance of 27 statements developed by the steering group [22] (see Tables 2 and 3) on a 9-point Likert scale. The statements were grouped under two headings, (Service Provision and Benefits of Integration) which were identified as being important to enhancing integration of diabetes and hypertension management with HIV management in SSA. For outcomes under the heading ‘Service Provision’ participants were asked to rate outcomes on a scale of 1–9 where 1 = lowest priority and 9 = highest priority. For outcomes under the heading ‘Benefits of Integration’ participants were asked to rate outcomes on a scale of 1–9 where 1 = strongly disagree and 9 = strongly agree. The Likert scale corresponds to the conventional format used for comparative assessment and prioritisation of different health options [24]. Participants could suggest additional outcomes during Round 1 by adding their suggested outcome(s) in a free text box. Due to time constraints for completing the study, participants were given a period of 1 week to complete round 1 of the survey and a reminder email was sent to those participants who had not yet completed the survey 2 days prior to the Round 1 deadline. Eighteen participants participated in Round 1 (with two partial completions, therefore 16 participants completed). Suggested outcomes from Round 1 were independently reviewed and coded by the first and second author to determine their novelty (i.e., that they were not covered by existing outcomes in the questionnaire). The first two authors could not reach agreement on whether to include two of the suggested outcomes, so clarification was sought from the last author (WC) and consensus was reached to include four of the 10 suggested outcomes in Round 2..

**Table 2:** Likert Scale scores for ‘Service Provision’ statements in Rounds 1 and 2

	Round 1				Round 2			
		Score 1-3	Score 4-6	Score 7-9		Score 1-3	Score 4-6	Score 7-9
Outcomes	n	n (%)	n (%)	n (%)	n	n (%)	n (%)	n (%)
Drug procurement systems for NCDs and HIV should be integrated	16	4 (25)	4 (25)	8 (50)	9	1 (11)	1 (11)	7 (78)
Drug procurement systems should be strengthened for NCDs for an integrated care programme	16	0 (0)	0 (0)	16 (100)	9	0 (0)	0 (0)	9 (100)
Patients with a chronic disease should be offered a choice of whether to collect routine medication from facilities or in the community	16	1 (6)	2 (12)	13 (82)	9	0	2 (22)	7 (78)
The same adherence interventions and adherence monitoring as used in HIV care should be applied to all patients with a chronic disease	16	1 (6)	3 (18)	12 (76)	9	1 (11)	1 (11)	7 (78)
There should be availability of equipment and access to relevant blood tests for routine monitoring for all conditions	16	0 (0)	0 (0)	16 (100)	9	0 (0)	0 (0)	9 (100)
Health education should be available for all chronic conditions within an integrated care clinic	16	0 (0)	0 (0)	16 (100)	9	0 (0)	0 (0)	9 (100)
There should be community-based education programs utilizing existing social, cultural and religious networks to proactively address stigma within NCD/HIV care	16	0 (0)	3 (18)	13 (82)	9	0 (0)	0 (0)	9 (100)
Most patients with multi-morbidity should be managed by non-physician health workers	16	6 (37)	4 (26)	6 (37)	9	5 (56)	1 (11)	3 (33)
There should be on-site training of health care workers on HIV and NCDs	16	0	3 (18)	13 (82)	9	0 (0)	0 (0)	9 (100)
An integrated care clinic should only deliver care to patients with multimorbidity	16	14 (88)	0 (0)	2 (12)	9	9 (100)	0 (0)	0 (0)
Task shifting should be an essential element of integrated care for HIV, hypertension and diabetes	16	0 (0)	4 (26)	12 (74)	9	0 (0)	0 (0)	9 (100)
Comprehensive community based NCD/HIV services should be used to propagate lifestyle modifications, adherence and follow up appointments so as to reduce the burden of complications and co-morbidities	16	0 (0)	1 (6)	15 (94)	9	0 (0)	0 (0)	9 (100)
Improved data collection and surveillance of NCDs among PLHIV should be used to inform integrated NCD/HIV programme management	16	0 (0)	0 (0)	16 (100)	9	0 (0)	0 (0)	9 (100)
It is important that referral networks from primary to secondary care are not adversely affected by integrated care delivery in primary care	16	1 (6)	4 (26)	11 (68)	9	0	1 (11)	8 (89)
It is important that problems with drug ordering and delivery do not undermine the capacity of ART sites to provide NCD care	16	0	1 (6)	15 (94)	9	0 (0)	0 (0)	9 (100)
^a^Integrated clinics may need to be re-launched to avoid being labelled as HIV clinics	16	NA	NA	NA	9	2 (22)	1 (11)	6 (67)
^a^All health workers should undergo training in the provision of integrated chronic care	16	NA	NA	NA	9	2 (22)	0 (0)	7 (78)

^a^Additional outcome suggested by a respondent in Round 1 and added for Round 2

**Table 3:** Likert Scale scores for ‘Benefits of Integration’ statements in Rounds 1 and 2

	Round 1				Round 2			
		Score 1-3	Score 4-6	Score 7-9		Score 1-3	Score 4-6	Score 7-9
Outcomes	n	n (%)	n (%)	n (%)	n	n (%)	n (%)	n (%)
Integrated services can enhance detection of HIV and its risk factors	16	3 (18)	5 (32)	8 (50)	9	2 (22)	3 (33)	4 (45)
Integrated services enhance detection of NCDs and their risk factors	16	2 (12)	2 (12)	12 (76)	9	1 (11)	1 (11)	7 (78)
Multi-morbidities (HIV, hypertension and/or diabetes) can be managed together at the same time and place by the same health care team of clinicians and nurses	16	0 (0)	2 (12)	14 (88)	9	0 (0)	1 (11)	8 (89)
Integrated services can be scaled-up within pre-existing structures	16	1 (6)	2 (12)	13 (82)	9	0 (0)	2 (22)	7 (78)
Integrated care will improve clinical outcomes for patients with multi-morbidities of HIV, hypertension and/or diabetes	16	0 (0)	1 (6)	15 (94)	9	0 (0)	1 (11)	8 (89)
An integrated clinic will provide better continuity of care for patients with multi-morbidities (HIV, diabetes and hypertension)	16	0 (0)	0 (0)	16 (100)	9	0 (0)	0 (0)	9 (100)
Integrated care for diabetes, hypertension and HIV can occur within the HIV care programme	16	1 (6)	3 (18)	12 (76)	9	0 (0)	3 (33)	6 (66)
Integrated care for HIV diabetes and hypertension can be delivered in the community	16	2 (12)	0 (0)	14 (88)	9	1 (11)	0 (0)	8 (89)
Integrated services may weaken the current HIV programme	16	9 (57)	4 (25)	3 (18)	9	8 (89)	0 (0)	1 (11)
Patients are likely to receive better quality of health education within the integrated clinic as within current vertical care clinics	16	0 (0)	5 (32)	11 (68)	9	0	4 (45)	5 (55)
Patients are likely to spend less time waiting in an integrated clinic	16	1 (6)	7 (44)	8 (50)	9	0 (0)	5 (55)	4 (45)
Patients with a chronic disease are more likely to be retained in an integrated care clinic	16	0 (0)	2 (22)	14 (78)	9	0 (0)	1 (11)	8 (89)
^a^Acceptability of integration of diabetes and hypertension with HIV may not be acceptable to all patients	0	NA	NA	NA	9	3 (33)	1 (11)	5 (55)
^a^The success of integrated care depends on how well the stakeholders work together	0	NA	NA	NA	9	0 (0)	1 (11)	8 (89)

^a^Additional outcome suggested by a respondent in Round 1 and added for Round 2

### Round 2

Round 2 included the 27 original outcomes, and four additional outcomes suggested by respondents in Round 1. The 16 participants, who had completed Round 1, were shown the distribution of Round 1 scores from other participants along with the score that they attributed to an outcome. Participants were asked to reflect on the score they had given to each statement, based on the scores of the other participants. Using the same 9-point Likert scale, they were invited to rescore if they wanted to. Participants were given 1 week to complete the second round of the survey. Through this process, consensus was reached on key components/outcomes, best practice, and likely benefits in integrated service provision for the prevention, identification and treatment of HIV, hypertension, and diabetes in SSA.

### Analysis

Data analysis was conducted using DelphiManager software. The DelphiManager software provided the user scores data for each statement. Consensus for each of the statements was defined a priori as 70% or more of the respondents scoring an outcome from seven to nine and fewer than 15% scoring it one to three. Meeting the consensus meant an outcome falling in both categories of threshold ≥70 and < 15%, The cut-off points were selected based on the most widely used cut of points in Delphi studies [18, 23, 25]. This would illustrate an outcome agreed as critically important by the majority and as of little or no importance by a small minority. Although there is no formal guidance for the reporting of e-Delphi studies, we followed recommendations including that patients and clinicians be involved; researchers and facilitators avoid imposing their views on participants; and attrition of participants be minimised as outlined by Sinha et al. [26].

### Ethical considerations

Ethical approval for the study was granted by the Human Research Ethics Committee at University College Dublin (LS-19-91-Cullen) and all methods were performed in accordance with the relevant guidelines and regulations. Participants were made aware that taking part in the study was optional and they could withdraw their participation at any time without reason. All anonymous information was securely stored on a password protected hard drive.

## Results

### Round 1

Of the 24 participants who were invited to take part in the study, 18 participated in Round 1 (6 of the invited participants did not commence Round 175% response rate). Participants’ ranking of outcome measures for Round 1 is provided in Tables 2 and 3.

Based on Round 1 ranking, 19 of the 27 outcomes for ‘Service Provision’ & ‘Benefits of Integration’ combined met the consensus threshold of ≥70% respondents scoring an outcome from seven to nine and < 15% scoring it one to three. (combined consensus rate =70.4%). With regards to the ‘Service Provision’ items, 11 outcomes met the consensus thresholds (consensus rate =73.4%), whilst 4 outcomes (consensus rate = 26.7%) did not. Of the 4 outcomes that did not meet thresholds, one item (*‘It is important that referral networks from primary to secondary care are not adversely affected by integrated care delivery in primary care’*) did meet the <15% threshold, but not the ≥70% requirement. Meanwhile, for the 12 Round 1 ‘Benefits of Integration’ items, 8 items met the thresholds (consensus rate =6.7%), and four did not (consensus rate = 33.4%) Of the items that did not meet the thresholds, two *(‘Patients are likely to receive better quality of health education within the integrated clinic as within current vertical care clinics’* and *‘Patients are likely to spend less time waiting in an integrated clinic’*) met the <15% threshold, but not the ≥ 70% criteria. Those statements for which all respondents scored 7–9 and statements for which most respondents scored 1–3 are illustrated in Tables 2 and 3.

### Round 2

Nine of Round 1’s 18 participants took part in and completed Round 2. Round 1’s participants suggested that four outcomes be added in Round 2, thus resulting in a new tally of 31 items for the combined measures. Two outcomes were added to the ‘*Service Provision*’ measure (‘*Integrated clinics may need to be re-launched to avoid being labelled as HIV clinics’* and ‘*All health workers should undergo training in the provision of integrated chronic care’*) and two were added to the ‘*Benefits of Integratio*n’ measure (‘*Acceptability of integration of diabetes and hypertension with HIV may not be acceptable to all patients’* and ‘*The success of integrated care depends on how well the stakeholders work together’*). Twenty-two of the 31 outcomes (consensus rate = 71.0%) met the ≥ 70 and <15% criteria. For the 15 items that were also on Round 1’s ‘*Service Provision*’ measure, 13 met the threshold criteria. Two items that did not meet the criteria in Round 1 did so in Round 2. These items were ‘*Drug procurement systems for NCDs and HIV should be integrated*’ and ‘*It is important that referral networks from primary to secondary care are not adversely affected by integrated care delivery in primary care*’. Meanwhile, of the two items that were added to Round 2, neither met the criteria. The second of these items ‘*All health workers should undergo training in the provision of integrated chronic care*’ did meet the ≥70% mark, but not the <15% threshold. As for Round 2’s ‘*Benefits of Integration*’ measure, 7 items (consensus rate = 58.4%) did meet the threshold criteria and 5 (consensus rate = 41.7%) did not. One item that met the criteria in Round 1 did not do so in Round 2. This item was ‘*Integrated care for diabetes, hypertension and HIV can occur within the HIV care programme’*. Of the two items that were added to Round 2’s ‘Benefits of Integration’ measure, one met the threshold criteria. This was the ‘ *… success of integrated care depends on how well the stakeholders work together’* measure.

Those statements for which all respondents scored 7–9 and statements for which most respondents scored 1–3 are illustrated in Tables 2 and 3. Those statements for which all respondents scored 7–9 on both rounds are illustrated in Tables 2 and 3.

## Discussion

This study sought to identify key components/outcomes and best practices in integrated service provision for the prevention, identification and treatment of HIV, hypertension, and diabetes. The results of our e-Delphi study suggest that key experts largely agree on what key components/outcomes and best practices should be involved when addressing the high and increasing dual burden of NCDs and HIV in sub-Saharan Africa.

The highest priority for service provisions was given to the strengthening of drug procurement systems for NCDs within an integrated care programme and the availability of equipment and access to relevant blood tests for routine monitoring for all conditions. Health education and community-based education programmes and services were also prioritised to address the stigma within NCD/HIV care. There should be onsite training of healthcare workers on HIV and NCDs. Participants also believed improved data collection and surveillance of NCDs among PLHIV should be used to inform integrated NCD/HIV programme management.

The results showed that participants strongly agreed that an integrated clinic would provide better continuity of care and clinical outcomes for patients with multi-morbidities. They believe patients with chronic diseases are more likely to be retained in an integrated care clinic and this care can be delivered in the community. Additionally, participants strongly disagreed that integrating these services may weaken the current HIV programme. One item that met the criteria in Round 1 did not do so in Round 2. This is a common occurrence with the Delphi consensus technique as participants are shown the distribution of Round 1 scores from other participants and asked to reflect on their own Round 1 score and are invited to rescore if they wanted to.

### How this relates to other literature

Although this topic has been studied before [27–29], the best practices in integrated care provision for the prevention, identification and treatment of hypertension, diabetes and HIV in SSA has yet to be identified. This study provides a unique perspective by a group of experts on the topic in order to inform best practice in integrated care provision. The results of this study reaffirm the view that an integrated clinic will provide better continuity of care for patients with diabetes, hypertension and HIV.

Barriers such as a lack of diagnostic equipment and medication [17, 30], lack of trained staff or training [30, 31] lack of guidelines and operating protocols [32], and perceived threat of integration to existing HIV success [33] have all been cited as issues when implementing integrated HIV/NCD care in SSA. These barriers were also highlighted by participants in this study as the need to strengthen [34] drug procurement systems for NCDs, improve the availability of equipment, and access to relevant blood tests for routine monitoring for all conditions were all strongly agreed upon. Health education and community-based education programmes and services were also prioritized.

The results of this study add to the existing literature by highlighting the most important service provisions and perceived benefits of integration, and particularly useful for the African context where integrated care is developing. This can be used as a guide to determining key outcomes and interventions in future trials.

### Limitations and strengths

This study employed the ‘Delphi’ consensus technique in an attempt to identify key components/outcomes and best practice in integrated service provision for the prevention, identification and treatment of HIV, hypertension, and diabetes. The validity of study findings depends on the composition of our e-Delphi panel. Recommended best practices in an e-Delphi study is to involve a diverse set of panellists [23]. The presence of diverse perspectives is likely to result in wider acceptance of the prioritized outcomes deemed important to include in future trials. To minimise recruitment bias, we invited a range of global and African stakeholder groups (researchers, policymakers, and academics) to participate in the study. While we could not control which stakeholders would return an expression of interest to participate in the study, the 24 expressions of interest and the nine participants who completed both rounds of the survey reflected a range of stakeholder groups and countries and, thus, captured a broad range of perspectives (see Table 1). The decision of 70% agreement could be a limitation considering Round 1 was only completed by 16 participants and Round 2 was only completed by nine participants, as there is some uncertainty as to what constitutes a consensus [26]. However, a recent systematic review [35] noted that few Delphi studies report response rates for all rounds and stated that the median number of invited participants was only 17 in Delphi studies. Therefore, we believe that our sample size is sufficient. Additionally, as our sample was chosen to be purposive, not representative, it can also be concluded that the decrease in participants from Round 1 to Round 2 is acceptable.

### Implications for research

Further research is needed to prioritise the outcomes identified in this study into a core outcome set to identify the best measures required to evaluate the benefits, challenges and cost-effectiveness of integration of HIV and NCD services in SSA.

## Conclusions

This study has identified the key components/outcomes that are most important to a range of key stakeholders in the field including researchers, policymakers, academics. While our Delphi panel included experts based outside SSA, we do not see this as being problematic as they were all experts on integrating diabetes, hypertension and HIV Care in SSA. The findings from this study will help guide future research when choosing key outcomes/interventions for future trials in this area and the 22 items prioritised here that met the ≥ 70 and <15% criteria will be useful to improve evidence synthesis in future systematic reviews. The identified key components are further essential to the generation of a culturally appropriate and transferable model of integration for potential operationalisation in Africa.

## Data Availability

The datasets used and/or analysed during the current study are available from the corresponding author on reasonable request.

## Abbreviations

ART: Antiretroviral therapy
LMIC: Low-to-middle-income-countries
NCD: Noncommunicable diseases
PLHIV: People living with HIV
SSA: Sub-Saharan Africa (SSA)
